# Supplementary material for: Inhalation of ACE2-expressing lung exosomes provides prophylactic protection against SARS-CoV-2
Source: Nat Commun. 2024 Mar 12;15:2236. doi: 10.1038/s41467-024-45628-x (PMC10933281; doi:10.1038/s41467-024-45628-x)
Supplement: Supplementary file 1 — Supplementary information [file 41467_2024_45628_MOESM1_ESM.pdf]

Inhalation of ACE2-expressing lung exosomes provides prophylactic protection against SARS-CoV-2

Wang et al.

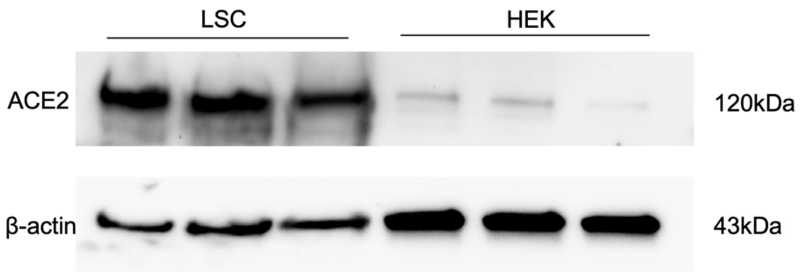

Fig. S1. Immunoblotting analysis of ACE2 expression in LSC and HEK cells. Uncropped blots are available in the Source Data file.

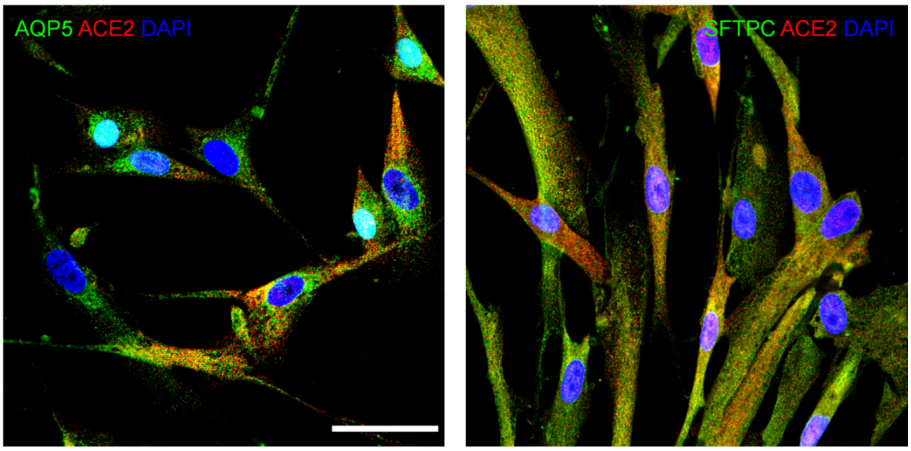

Fig. S2. Confocal images of LSC labelled with ACE2 (red), AQP5 (green) and SFTPC (green) antibodies. 4′,6-diamidino-2-phenylindole (DAPI, blue) was used to show nuclei. Scale bar: 50 µm.

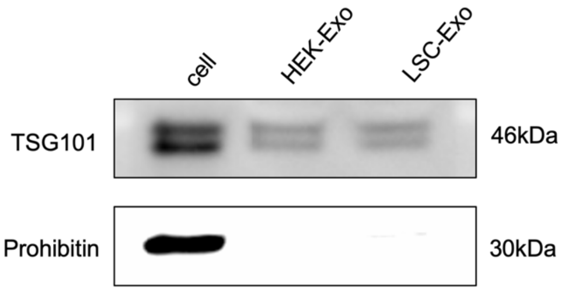

Fig. S3. Western blot of TSG101 and Prohibitin expression on HEK293T cells, HEK-Exo and LSC-Exo. Uncropped blots are available in the Source Data file.

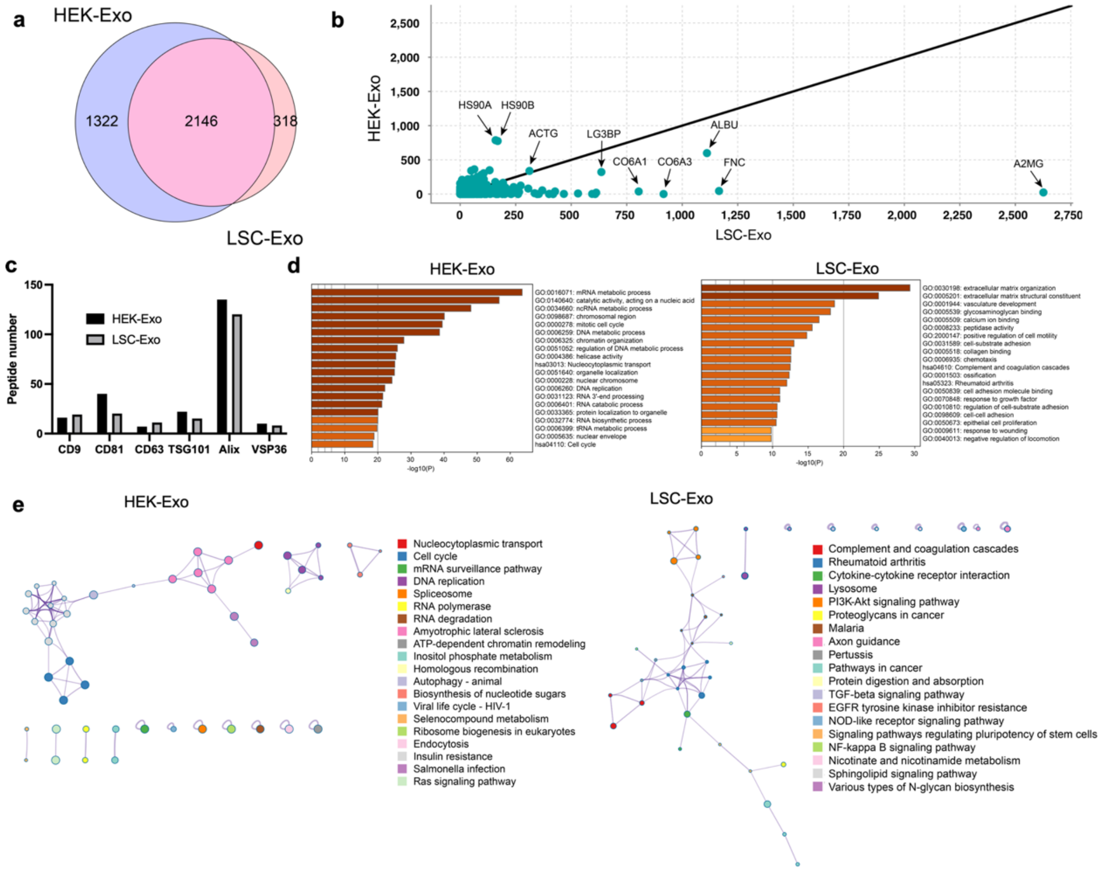

Fig. S4. Proteomics analysis of LSC-Exo and HEK-Exo. (a) Venn diagram of proteins identified in LSC-Exo and HEK-Exo. (b) Correlation scatterplots analysis of shared proteins in LSC-Exo and HEK-Exo. (c) Peptide numbers of specific biomarkers of HEK-Exo and LSC-Exo. Source data are provided as a Source Data file. (d) GO function analysis of LSC-Exo and HEK-Exo. (e) KEGG pathway enrichment of LSC-Exo and HEK-Exo.

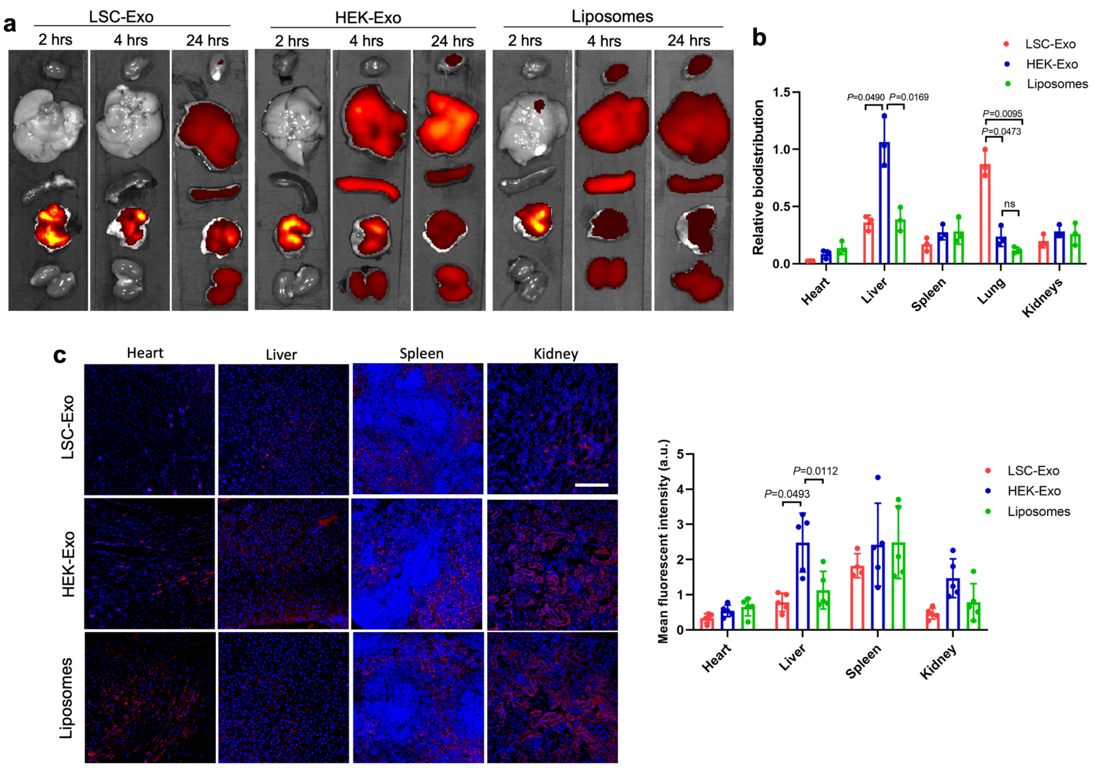

Fig. S5. Biodistribution of LSC-Exo in mice after inhalation. (a) Ex vivo imaging of major organs of mice after RFP-LSC, RFP-HEK or RFP-Lipo inhalation at the indicated time. (b) Quantification of the integrated density of RFP fluorescence in major organs; *n*=3 per group. (c) Confocal images showing the biodistribution of RFP-LSC, RFP-HEK or RFP-Lipo in heart, liver, spleen and kidney tissues and quantitative results from heart, liver, spleen and kidney tissues. Scale bar, 50 µm. *n*=5 per group. Data are mean ± s.d. Statistical analysis was performed by two-way ANOVA with Tukey's multiple comparisons. Source data are provided as a Source Data file.

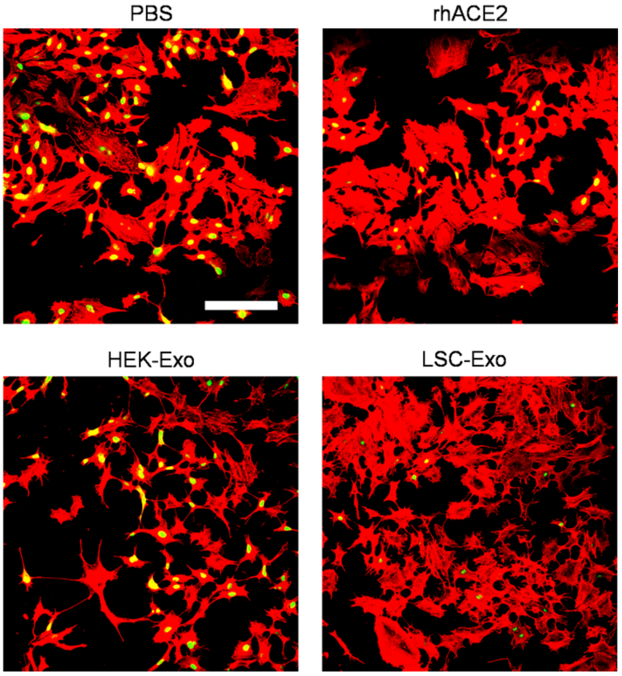

Fig. S6. Representative confocal imaging of A549 cells expressing ACE2 infected with SARS-CoV-2 pseudovirus, which inhibited by LSC-Exo or HEK-Exo or rhACE2 treatment. Phalloidin (red) and SARS-CoV-2 pseudovirus (green). Scale bar: 100 µm.

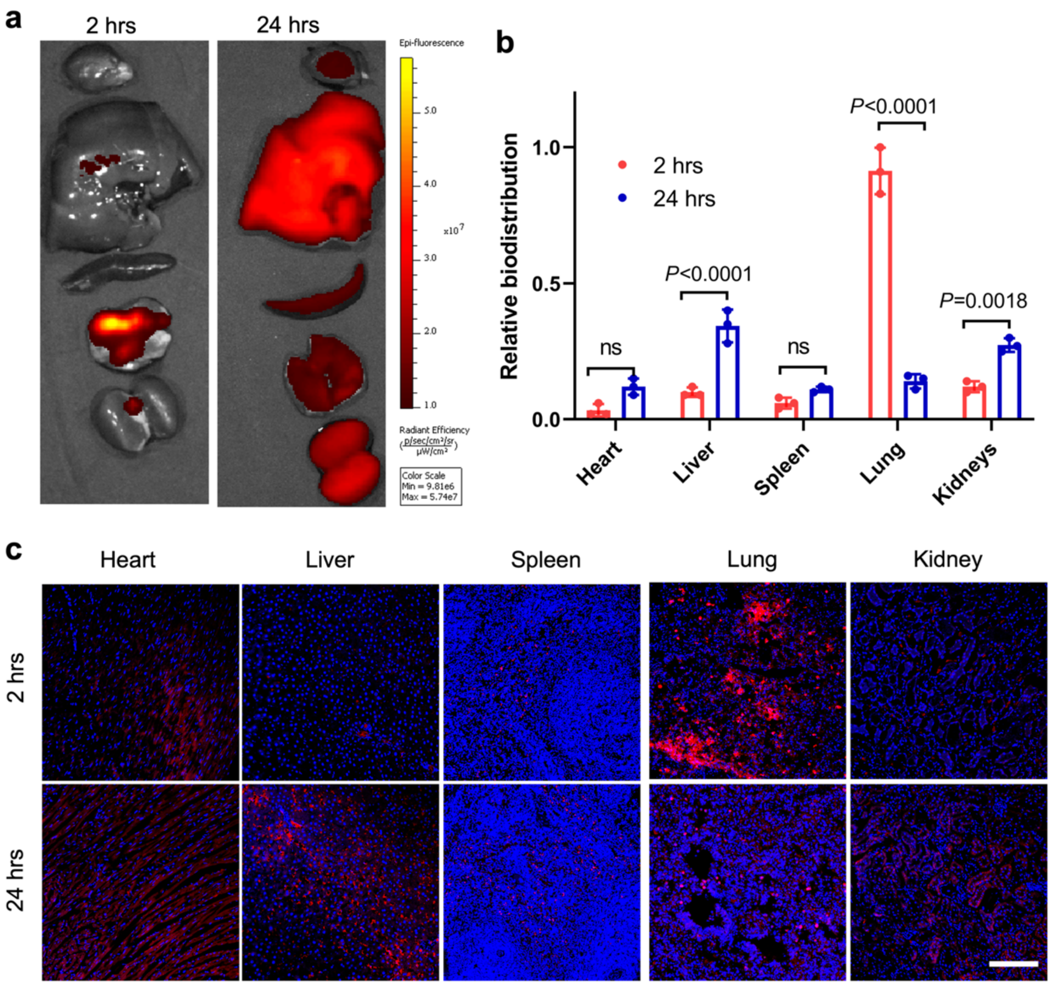

Fig. S7. Biodistribution of LSC-Exo in hamsters after inhalation. (a) Ex vivo imaging of major organs of hamsters 2 hours and 24 hours after RFP-LSC inhalation. (b) Quantification of the integrated density of RFP fluorescence in major organs; *n*=3 per group. Source data are provided as a Source Data file. (c) Confocal images showing the biodistribution of LSC-Exo in heart, liver, spleen, lung and kidney tissues of hamsters. Scale bar, 50 µm. Data are mean ± s.d. Statistical analysis was performed by two-way ANOVA with Tukey's multiple comparisons.

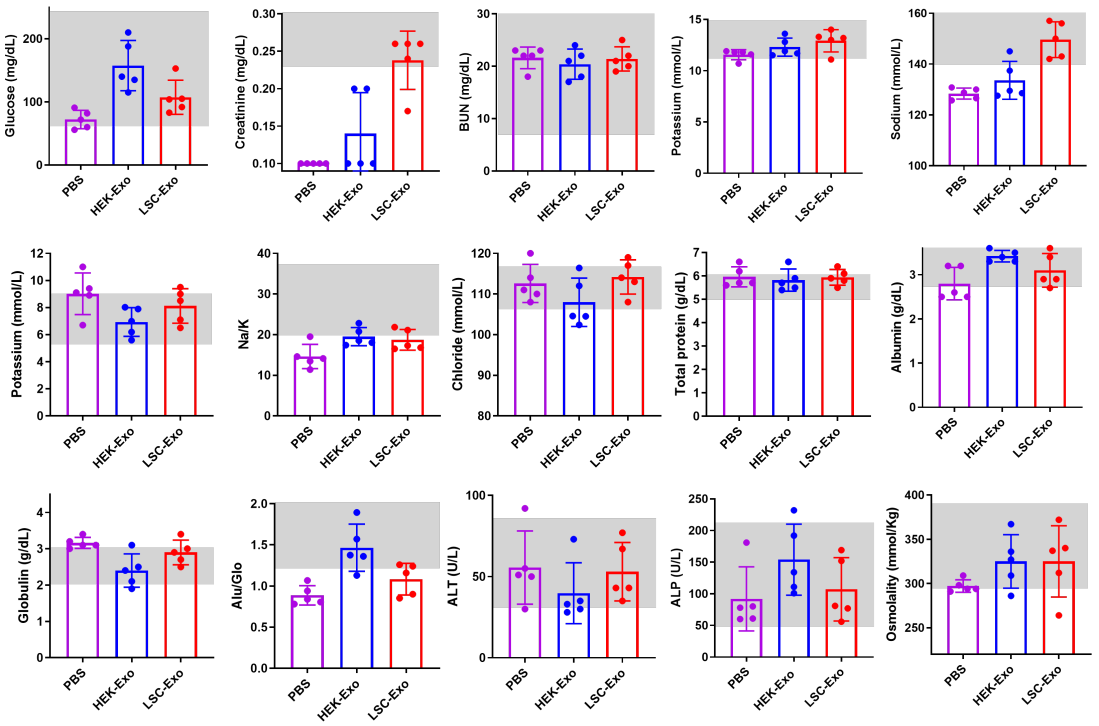

Fig. S8. Clinical chemistry parameters from the peripheral blood of hamsters 7 days post authentic SARS-CoV-2 challenge. Each dot represents data from one animal. Data are mean ± s.d. *n*=5. The grey area represents the normal ranges of these parameters in hamsters. Source data are provided as a Source Data file.

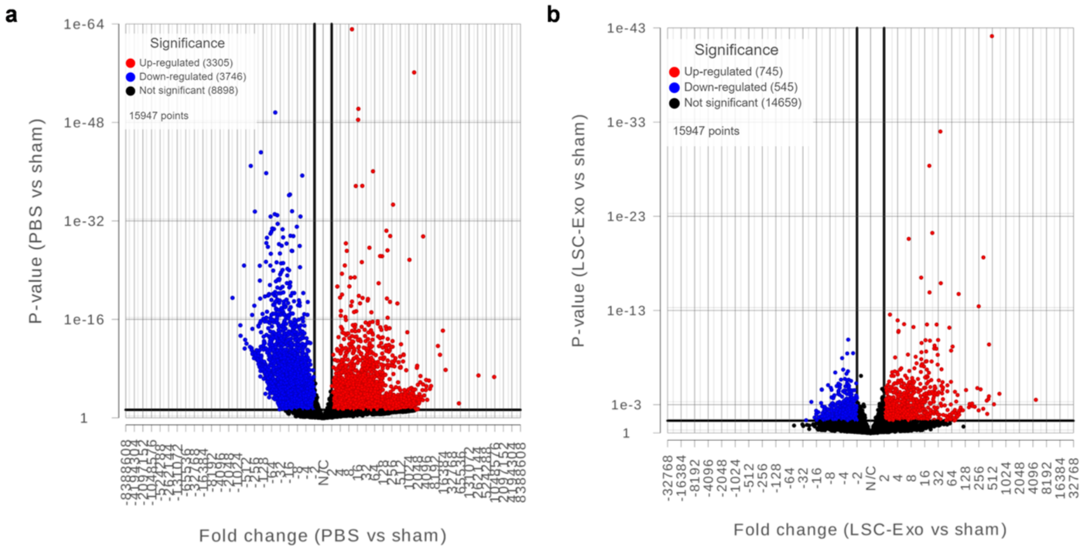

Fig. S9. Volcano plots comparing differentially expressed genes from lung tissues of hamsters with different treatments. (a) Comparison of PBS group versus sham. (b) Comparison of LSC-Exo group vs sham. Red and blue indicate upregulated genes and

downregulated genes with a fold change > 1 and P_{adj}<0.05, respectively. *n*=3 for PBS group and *n*=4 for LSC-Exo group.

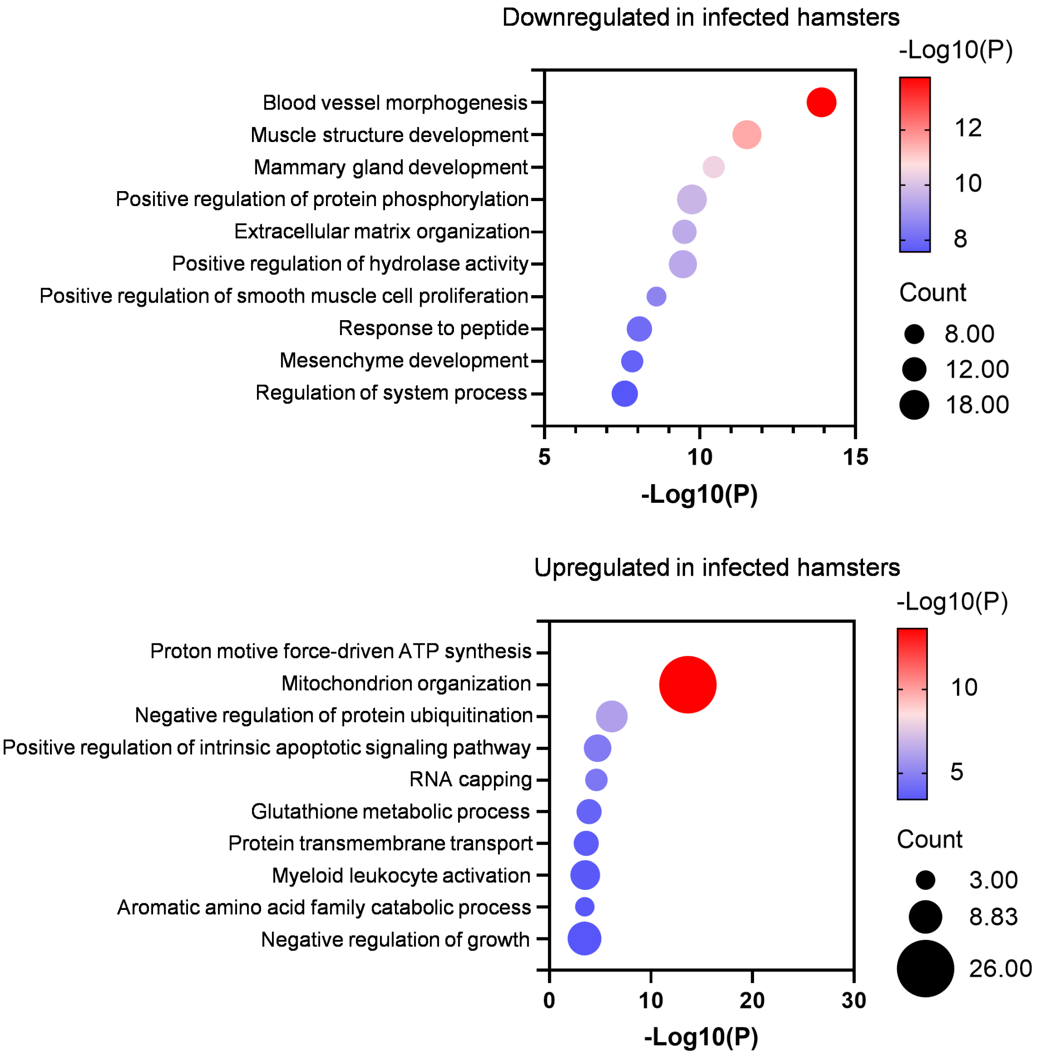

Fig. S10. GO enrichment analysis of biological process terms enriched in downregulated and upregulated genes from comparisons of infected hamsters versus sham hamster.

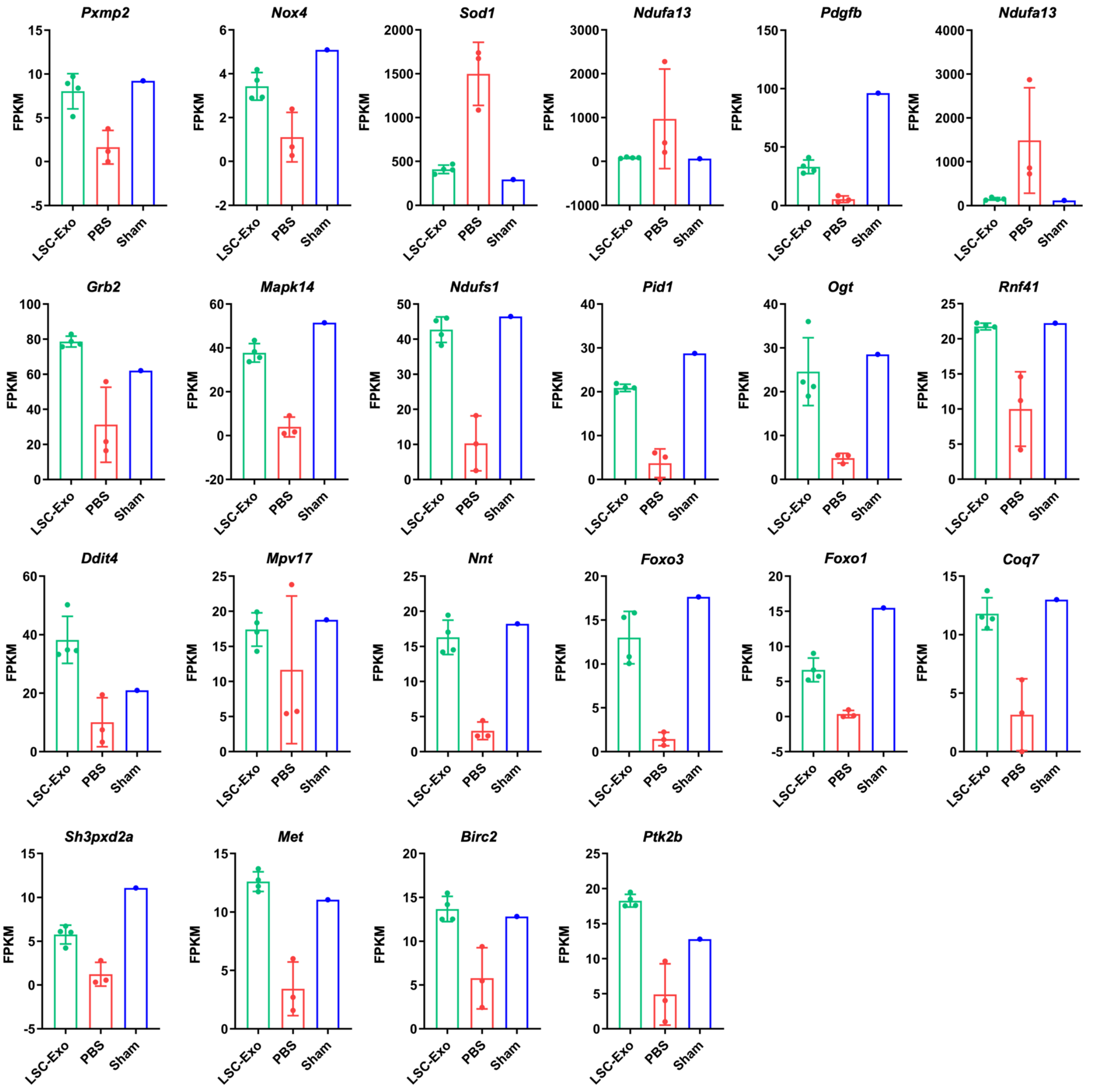

Fig. S11. RNA-Seq results expressed as Fragments per kilobase of exon per million mapped reads (FPKM) for genes related to regulation of ROS metabolic process. Each dot represents data from one animal. *n*=3 for PBS group and *n*=4 for LSC-Exo group. Data are mean ± s.d. Source data are provided as a Source Data file.

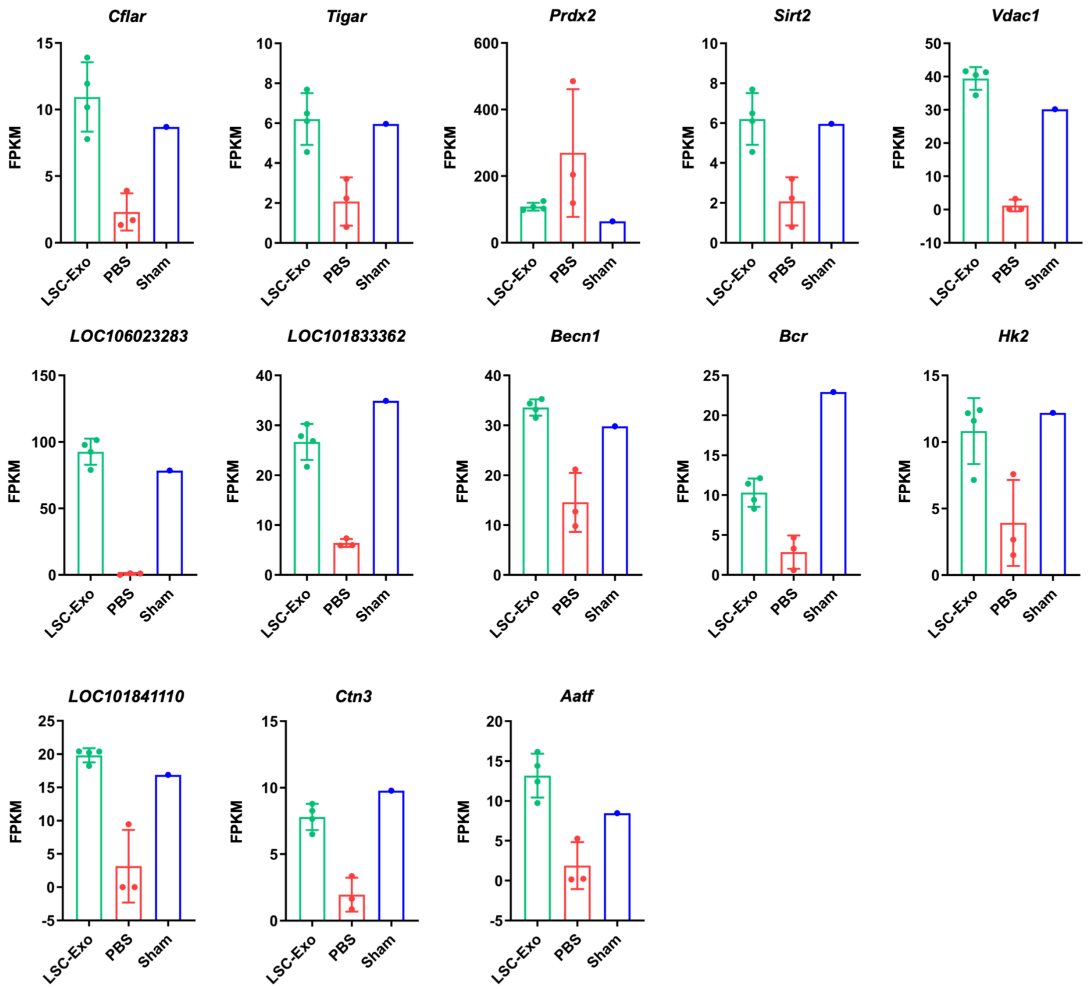

Fig. S12. RNA-Seq results expressed as FPKM for genes related to negative regulation of ROS metabolic process. Each dot represents data from one animal. *n*=3 for PBS group and *n*=4 for LSC-Exo group. Data are mean ± s.d. Source data are provided as a Source Data file.

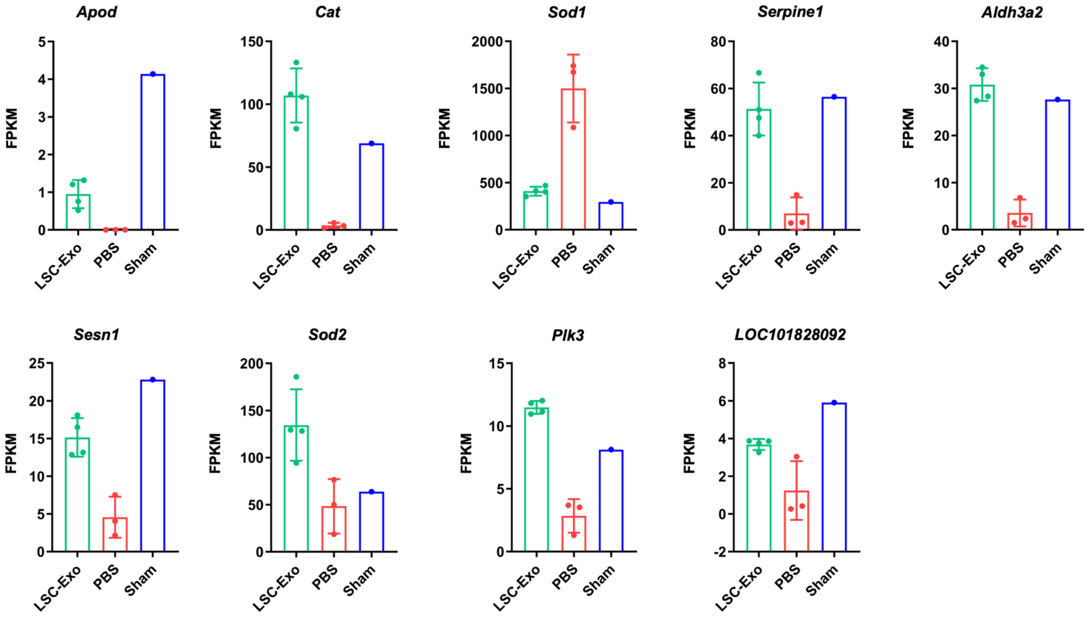

Fig. S13. RNA-Seq results expressed as FPKM for genes related to response to ROS. Each dot represents data from one animal. *n*=3 for PBS group and *n*=4 for LSC-Exo group. Data are mean ± s.d. Source data are provided as a Source Data file.

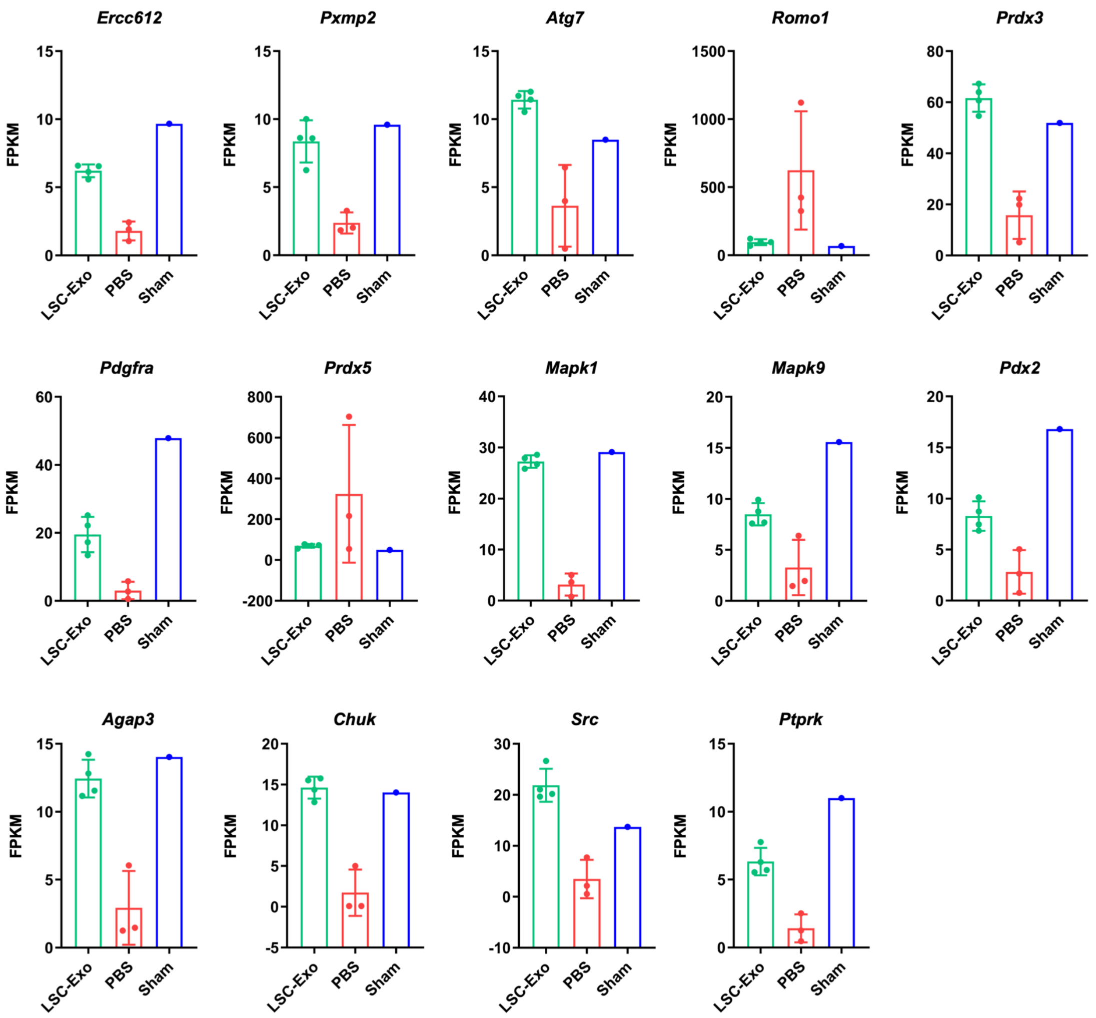

Fig. S14. RNA-Seq results expressed as FPKM for genes related to cellular response to ROS. Each dot represents data from one animal. n=3 for PBS group and n=4 for LSC-Exo group. Data are mean ± s.d. Source data are provided as a Source Data file.

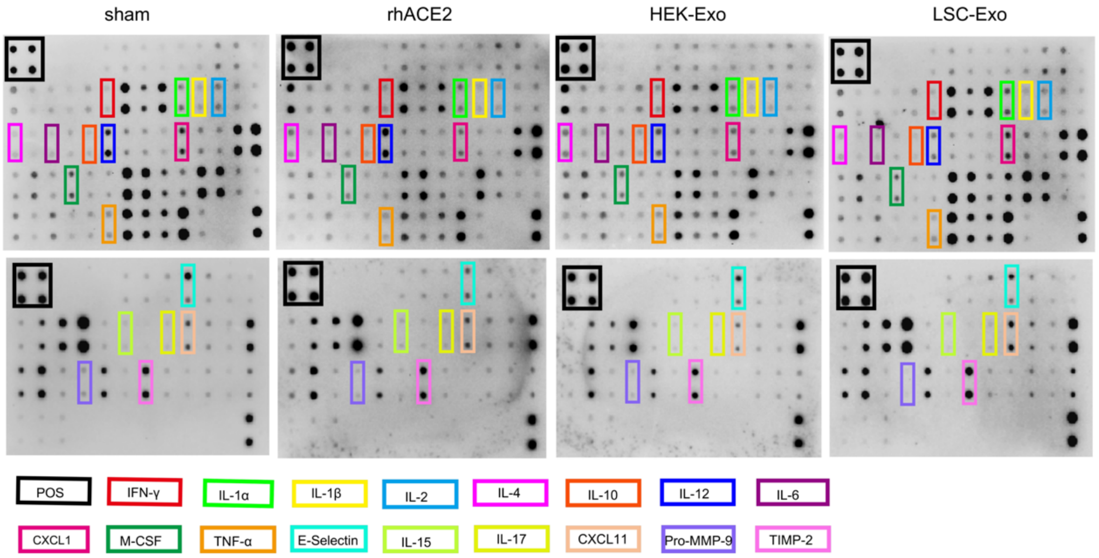

Fig. S15. Cytokine array to determine inflammatory cytokines from mice serum 7 days after rhACE2 or HEK-Exo or LSC-Exo inhalation.

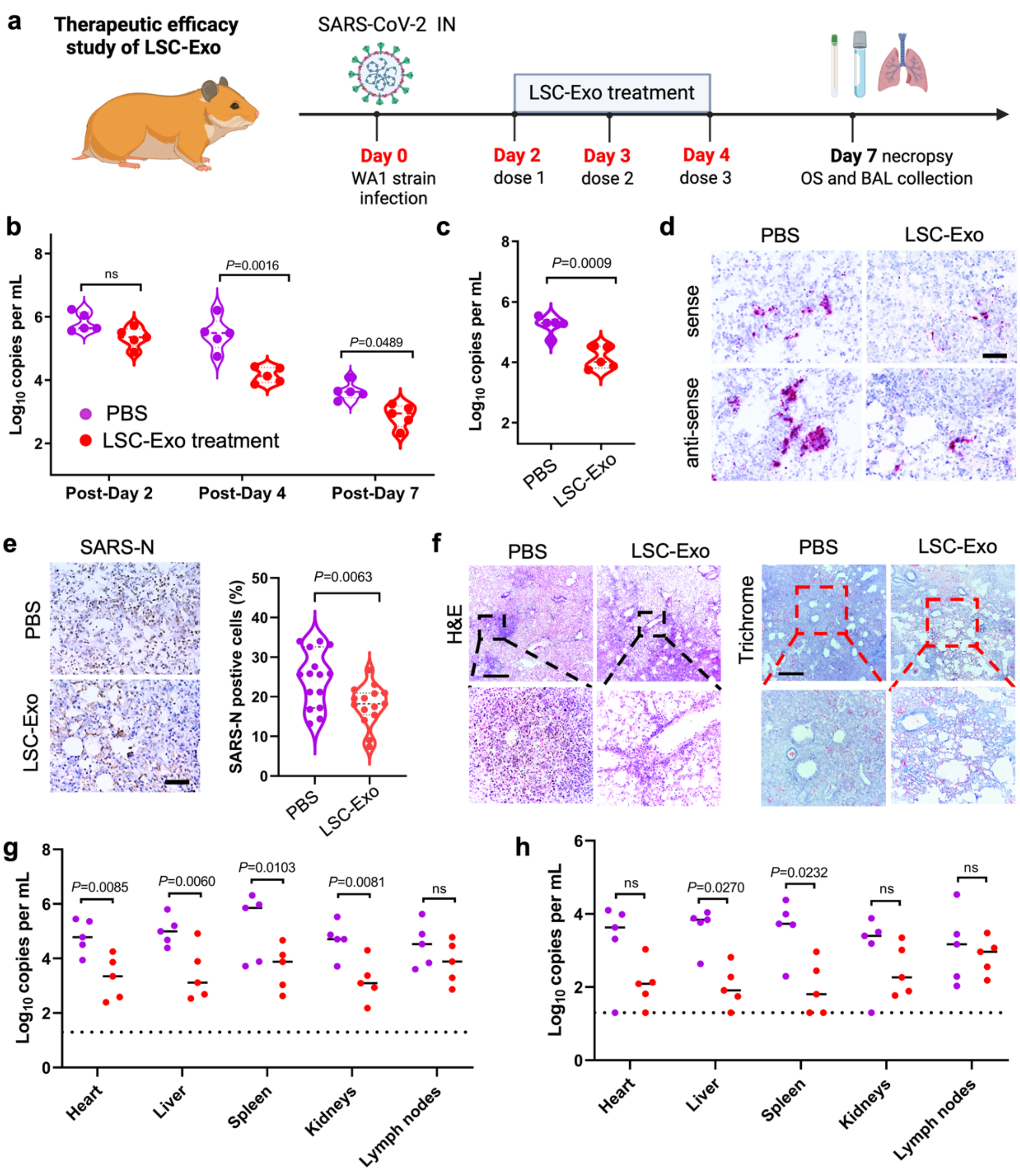

Fig. S16. Therapeutic efficacy of LSC-Exo against original SARS-CoV-2 WA1 infection in hamsters. (a) Study design of LSC-Exo as a therapeutic agent against SARS-CoV-2 infection, created with Biorender.com. (b) Viral RNA in oral swabs from hamsters treated with LSC-Exo or PBS. *n*=5. (c) Viral RNA in bronchoalveolar lavage (BAL) fluid from hamsters treated with LSC-Exo or PBS at 7 days post-challenge. *n*=5. Data are mean ± s.d. (d) RNAscope images of hamster lungs. Scale bar, 50 µm. (e) Immunohistochemistry analysis and quantification analysis of SARS-N protein in lung tissues of hamsters. Scale bar, 50 µm. *n*=15 (f) H&E staining and Masson's trichrome images of lung sections of hamsters. Scale bar, 50 µm. Viral genomic RNA levels (g) and sgRNA levels (h) in tissues of hamsters with LSC-Exo or PBS. *n*=5. Data are

mean ± s.d. Statistical analysis was performed by two-way ANOVA with Tukey's multiple comparisons (b, g and h) or two-tailed, unpaired Student's *t*-test (c and e). The data from ***the PBS group presented here in panels (b-h) are recreated from the PBS group in Fig. 4.*** Source data are provided as a Source Data file.

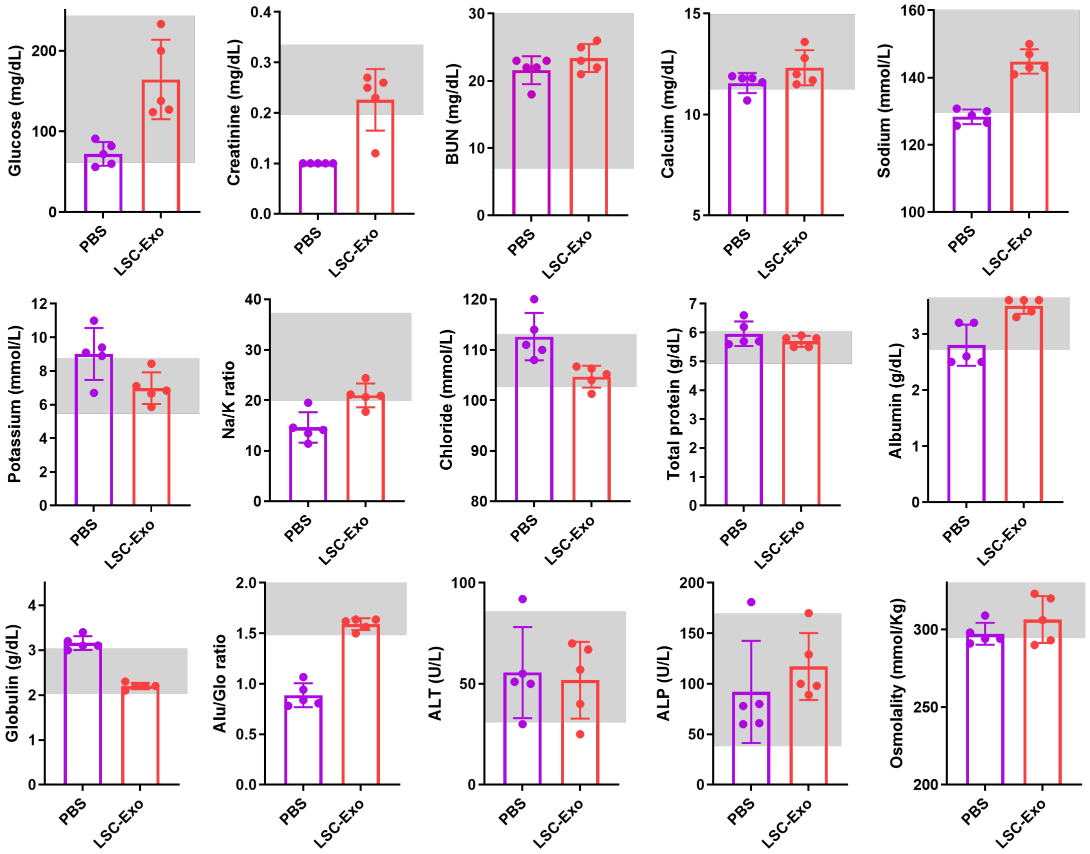

Fig. S17. Clinical chemistry parameters from the peripheral blood of hamsters 7 days post authentic SARS-CoV-2 challenge. Each dot represents data from one animal. Data are mean ± s.d. n=5. The grey area represents the normal ranges of these parameters in hamsters. Source data are provided as a Source Data file.

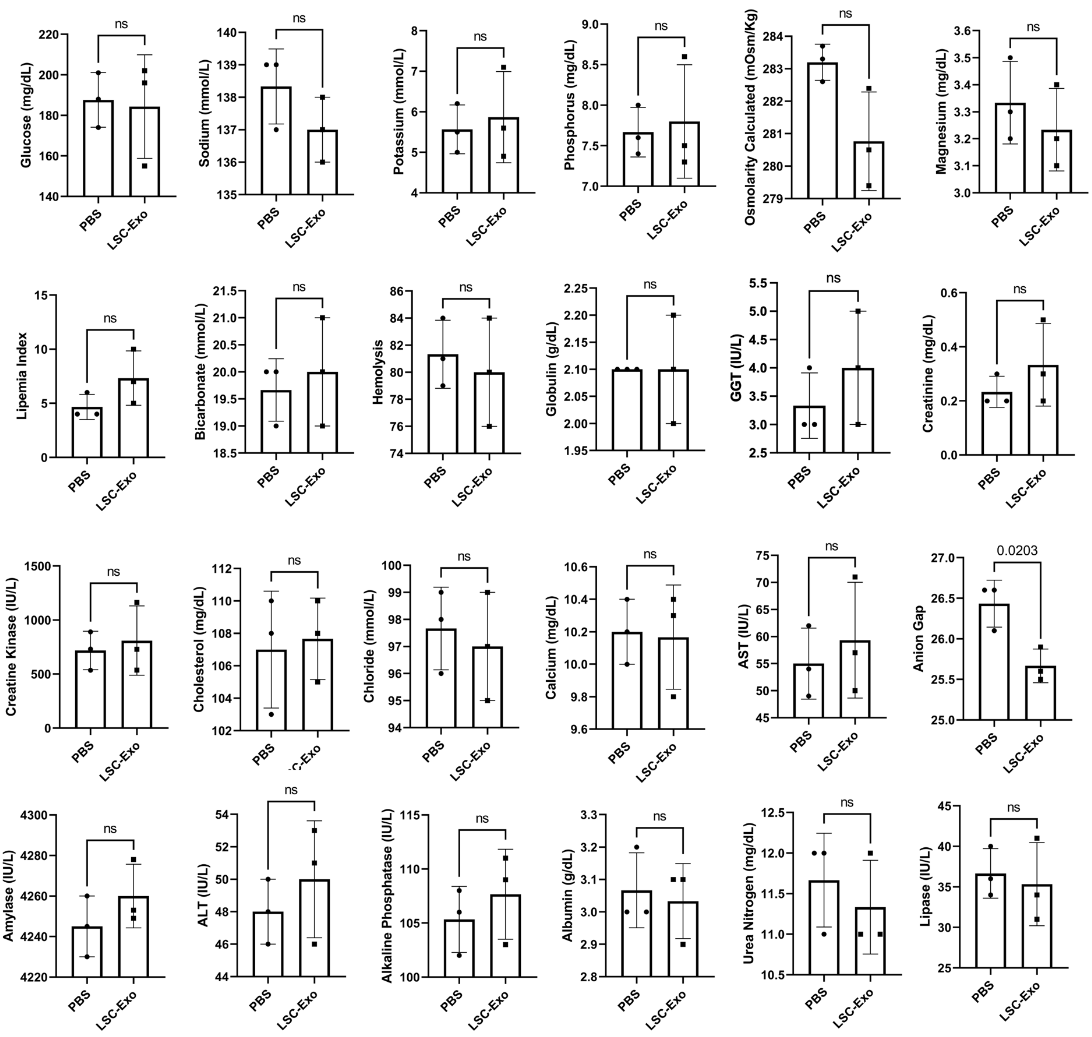

Fig. S18. Clinical chemistry parameters from the peripheral blood of hamsters 7 days after LSC-Exo inhalation. Each dot represents data from one animal. Data are mean ± s.d. *n*=3. Statistical analysis was performed by the two-tailed, unpaired Student's *t*-test. Source data are provided as a Source Data file.

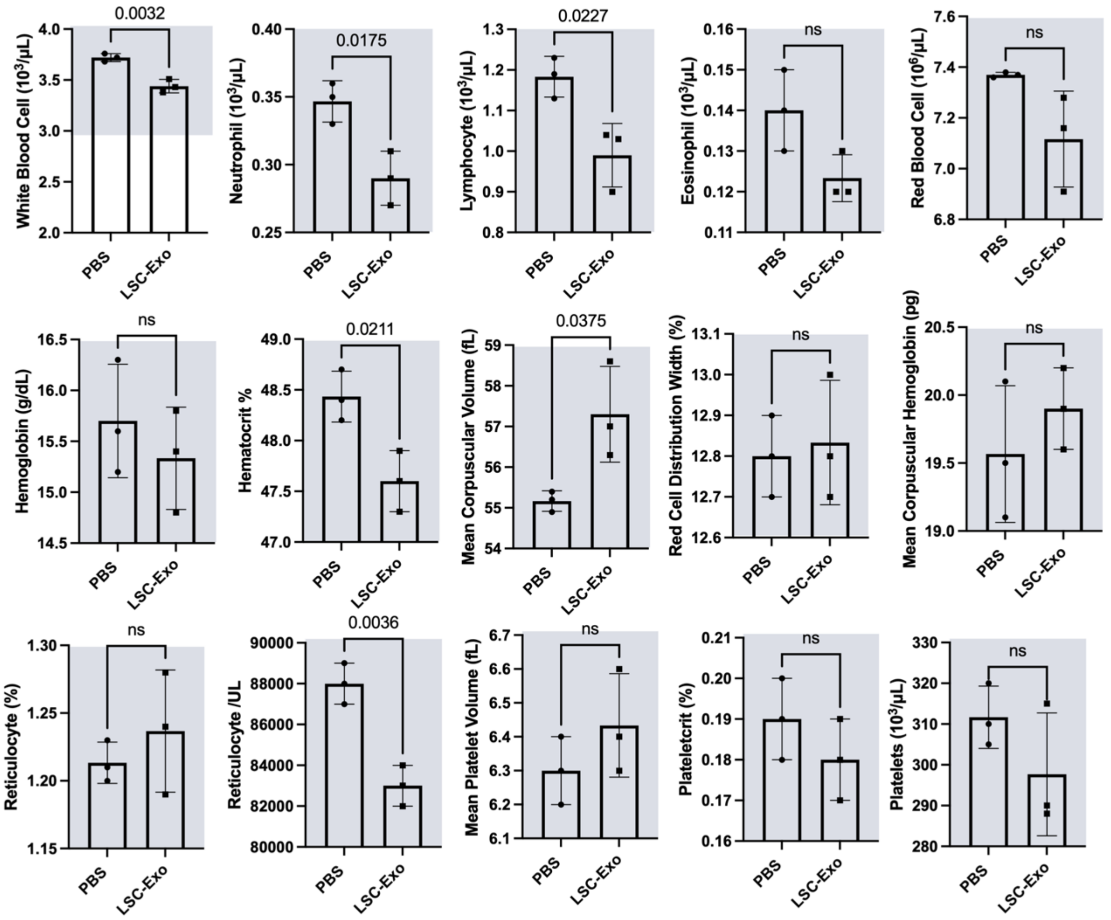

Fig. S19. Complete blood count analysis from the peripheral blood of hamsters 7 days after LSC-Exo inhalation. Each dot represents data from one animal. Data are mean ± s.d. *n*=3. Statistical analysis was performed by the two-tailed, unpaired Student's *t*-test. Source data are provided as a Source Data file.

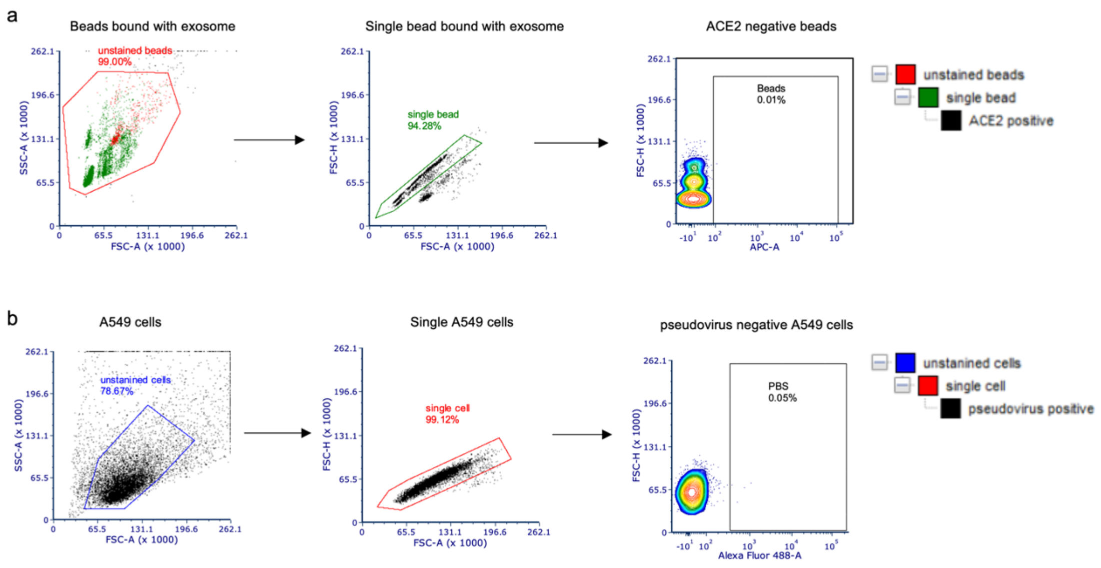

Fig. S20. Gating strategies used for flow cytometry assay. (a) Gating strategy to detect the expression of ACE2 receptor on LSC-Exo and HEK-Exo in Fig. **1f.** (b) Gating strategy to detect the A549 cells infected by SARS-CoV-2 pseudovirus or SARS-CoV-2 VOC pseudoviruses in Figs. **3f** and **6a**.
